# Toward an integrative account of social cognition: marrying theory of mind and interactionism to study the interplay of Type 1 and Type 2 processes

**DOI:** 10.3389/fnhum.2012.00274

**Published:** 2012-10-11

**Authors:** Vivian Bohl, Wouter van den Bos

**Affiliations:** ^1^Department of Philosophy, Institute of Philosophy and Semiotics, University of TartuTartu, Estonia; ^2^Center for Subjectivity Research, University of CopenhagenCopenhagen, Denmark; ^3^Decision Neuroscience Lab, Department of Psychology, Stanford UniversityStanford, CA, USA

**Keywords:** social cognition, social interaction, theory of mind, interactionism, dual process theories, Type 1 processes, Type 2 processes

## Abstract

Traditional *theory of mind* (ToM) accounts for social cognition have been at the basis of most studies in the social cognitive neurosciences. However, in recent years, the need to go beyond traditional ToM accounts for understanding real life social interactions has become all the more pressing. At the same time it remains unclear whether alternative accounts, such as *interactionism*, can yield a sufficient description and explanation of social interactions. We argue that instead of considering ToM and interactionism as mutually exclusive opponents, they should be integrated into a more comprehensive account of social cognition. We draw on *dual process models* of social cognition that contrast two different types of social cognitive processing. The first type (labeled *Type 1*) refers to processes that are fast, efficient, stimulus-driven, and relatively inflexible. The second type (labeled *Type 2*) refers to processes that are relatively slow, cognitively laborious, flexible, and may involve conscious control. We argue that while interactionism captures aspects of social cognition mostly related to Type 1 processes, ToM is more focused on those based on Type 2 processes. We suggest that real life social interactions are rarely based on either Type 1 or Type 2 processes alone. On the contrary, we propose that in most cases both types of processes are simultaneously involved and that social behavior may be sustained by the interplay between these two types of processes. Finally, we discuss how the new integrative framework can guide experimental research on social interaction.

## Introduction

In the past three decades, intensive discussions on social cognition have taken place in philosophy and in empirical sciences. Until recently, the so called *theory of mind* (ToM) approach (understood as encompassing *theory theory* (TT), *simulation theory* (ST), and their hybrids) has been the dominant theoretical framework. However, the situation has started to change, partly due to extensive critique of ToM by authors who draw on phenomenological, enactive, and embodied approaches to cognition (see e.g., Hutto and Ratcliffe, 2007; Zlatev et al., 2008; Leudar and Costall, 2009b; De Jaegher et al., 2010). These critics have pointed out that in order to understand real life social interactions we need to go beyond traditional accounts of social cognition. Nevertheless, because the interactionists do not offer a clear and unified alternative paradigm, it remains unclear whether the alleged interactionist alternative (understood broadly as a set of approaches to social cognition that insist on replacing the traditional ToM paradigm by a new theoretical framework focusing on embodied and supra-individual aspects of real life social interactions) can yield a sufficient description and explanation of social interactions and of social cognition in general.

The aim of this paper is to argue that ToM and interactionism ought not to be considered as mutually exclusive opponents. Instead, they should be integrated into a single comprehensive theoretical framework for understanding social cognition. In order to develop new research questions and hypotheses from the integrative ToM-interactionist framework, we draw upon dual process theories of social cognition that contrast two different types of social cognitive processing. The first type (labeled *Type 1*) refers to processes that are fast, efficient, stimulus-driven, and relatively inflexible. The second type (labeled *Type 2*) refers to processes that are slow, cognitively laborious, flexible, and may involve consciousness. We argue that while interactionism captures types of phenomena of social behavior mostly related to Type 1 processes, ToM is more focused on those based on Type 2 processes. We suggest that real life social interactions are rarely based on either Type 1 or Type 2 processes alone; on the contrary, we propose that in most cases both processes are simultaneously involved and that social behavior moreover may be sustained by the interaction between these two types of processes. From this line of reasoning a new challenge for the research of social cognition emerges: the need to study the interplay between Type 1 and Type 2 processes in social interactions.

The paper is divided into several parts. In section “Theory of Mind and Interactionism” we introduce the theoretical framework of the traditional ToM accounts and outline the interactionist alternative to ToM. Next, in section “ToM or Interactionism? Both!”, we argue that ToM and interactionism address different aspects of social cognition and should therefore not be considered as mutually exclusive paradigms, but rather as complementary. This is followed by a brief overview of the dual process accounts in section “The Dual Models of Cognition: Type 1 and Type 2 Processes”. Here we introduce the dual process theories of social cognition that have been proposed in social psychology and social neuroscience, and we draw parallels between the Type 1 and Type 2 processes on the one hand, and the interactionist and ToM accounts on the other. In “Joining Theory of Mind and Interactionism to Study Dual Processes” we propose that marrying ToM and interactionism for studying different types of social cognitive processes leads to building a new comprehensive theoretical framework that raises new interesting research questions and hypotheses. In “An Integrative Framework” we outline a new hypothesis, based on the integrative account, that Type 1 and Type 2 processes can have mutual effects upon each other during social interactions. Finally, in section “Theoretical Concerns Become Methodological Concerns”, we address some of the methodological issues that follow from this new approach and discuss how the integrative framework can guide experimental research on social interaction.

## Theory of mind and interactionism

### Theory of mind

The framework of ToM encompasses two theories: TT and ST. There are several versions of both of them, as well as hybrid versions that combine the two. The central claim of ToM is that we understand other people's behavior by attributing mental states to them.

According to TT, our ability to attribute mental states to others as well as to ourselves relies on the use of a specific theory—folk psychology. Folk psychology postulates mental states (beliefs, intentions, desires, pains, fears, etc.) as theoretical entities that are bound together in commonsensical principles and generalizations, enabling us to explain and predict observable behavior in mental terms, i.e. to “mindread” or “mentalize”^1^. The mastering of folk psychological theory on some level of cognition is taken to be the fundamental mechanism of social cognition. There are explicit and implicit versions of TT depending on whether the use of theory is claimed to be explicit or whether the understanding of others is considered implicitly structured in the form of a theory. Some theory theorists have defended the idea of “the child as scientist” where our mental concepts are successively learned and replaced in development in analogous manner to scientific change (Astington and Gopnik, 1988; Wellmann, 1990; Gopnik and Meltzoff, 1997; for criticism of this view see Bishop and Downess, 2002). Others have favored the idea of modularity (Fodor, 1983), arguing that humans have an innate cognitive module for mindreading (possibly constituted by several sub-modules) which is separate from the general intellectual capacities and “ripens” during childhood (Baron-Cohen et al., 1985; Leslie, 1994; Baron-Cohen, 1995).

According to ST, there is no need for mastering the folk-psychological theory, since we use our own minds as models for understanding other minds. We apprehend the mental states of others by simulating them with the help of our own cognitive mechanisms. There are various versions of ST. Simulation can either be taken to be a conscious use of imagination and inference, i.e., mentally putting oneself in the “shoes” of the other (Goldman, 1989), an explicit but non-inferential process (Gordon, 1995), an implicit and sub-personal process (Gallese and Goldman, 1998), or a combination of implicit and explicit simulation (Goldman, 2006). While the majority of simulation accounts aim to make empirical claims, Jane Heal has defended a simulation account of an *a priori* nature, claiming that thinking about other minds necessarily involves recreation of the other's point of view (Heal, 1998).

As stated above, various authors have also argued in favor of hybrid versions that combine theorizing and simulation in order to overcome the problems that each of these theories face by themselves (e.g., Nichols and Stich, 2003; Goldman, 2006).

### Common assumptions of ToM under criticism

Although TT and ST have been developing as rivals, they share several fundamental assumptions and methodological strategies (which is why we discuss them under the common label “theory of mind”)^2^. In the course of the last decade, the common assumptions have been criticized by e.g., Gallagher (2001, 2007, 2008b), Gallagher and Zahavi (2008), Hutto (2004), Reddy (2008), Reddy and Morris (2004), Leudar and Costall (2009a), De Jaegher (2009a,b), De Jaegher and Di Paolo (2007, 2008), De Jaegher et al. (2010), and Zahavi (2007, 2008, 2011). Our aim here is not to reconstruct every argument against ToM that each author has provided; rather we briefly elucidate the common assumptions of the ToM accounts that most of these criticisms have pointed out.

According to interactionists, one of the core implications, no matter which version of TT, ST, or their hybrids we consider, is the denial of the possibility that we can directly grasp other people's mental states (see e.g., Reddy, 2008; Gallagher and Zahavi, 2008). In order to understand others, an explicit or implicit process of theorizing or simulation is claimed to be necessary, because mental states are typically conceptualized as “unobservable”^3^. Interactionists reject this implication, insisting that we seldom see bare movements of human bodies; rather, the actions of others are already perceived as intelligent behavior (see Zahavi, 2007; Gallagher, 2008b)^4^.

Another criticized assumption is that of “third-person mindreading” being essential for social cognition (see e.g., Gallagher, 2008a). This assumption has two components: the “third-person” component and the “mindreading” component. The “third-person” assumption holds that theories designed to explain observational mindreading from a third-person point of view are adequate for explaining social understanding in general, including interpersonal understanding from a participatory “second-person” point-of-view. The “mindreading” assumption holds that mindreading (attribution of mental states to explain or predict behavior) is pervasive in social cognition. According to interactionism, ToM accounts erroneously assume a rather strong cocktail of both components, suggesting that the “third-person mindreading” is first and foremost what social cognition is about.

A final common feature to all ToM approaches is that they focus on cognitive processes of individuals and pay little attention to other enabling factors of social cognition, such as context, environment, and embodiment. The aim of ToM is to explain what cognitive processes are responsible for human social cognition. This aim has been criticized as being excessively individualist and overly cognitivist because it underestimates the supra-individual^5^ and embodied aspects of social cognition.

These common assumptions have had strong influence on the empirical methodology that has been used for testing the empirical hypotheses of ToM. For example, in the classical false-belief tasks, test subjects are asked to interpret others' behavior by merely looking at a scene as passive observers; they do not interact with the characters they observe. Interactionists have pointed out that observational mindreading tasks reveal little about the processes that are important for participatory social interactions^6^.

We will discuss some of the assumptions of ToM to a greater extent in the next section where we introduce the interactionist position as based on a critique against ToM.

### Interactionism in contrast to ToM

While standard ToM approaches rarely emphasize social interaction, it is the heart of the approach that we ascribe to “interactionists”: “[w]hat we call social cognition is first of all social interaction” (Gallagher, 2008a). Recently, John Michael (2011) has defined interactionism as referring to a family of positions endorsing the claim that “social understanding and interaction do not require mindreading because various embodied and/or extended capabilities sustain social understanding and interaction in the absence of mindreading” (559–560). We will take this definition as our starting point. However, in order to get a better grip on what interactionism amounts to, we will look more closely at how it differs from and contrasts with ToM.

#### Against cognitivism and individualism

The position of interactionists can be characterized as anti-individualist^7^ and anti-cognitivist: social interaction cannot be fully explained by referring to processes evolving in the minds (or brains) of individuals while they infer or simulate the unobservable mental states of other individuals. In order to understand social interaction, it is necessary to look at the interaction process on the supra-individual level as something evolving *between* subjects (Reddy and Morris, 2004, p. 653) and as something that is not reducible to each individual's cognitive input to the process (De Jaegher et al., 2010). According to interactionism, mindreading can be considered superfluous for social interaction in many real-life pragmatic contexts, because the interaction process can be more adequately characterized as participants making sense of the situation together, rather than participants reacting to each-other via the “double-screen” of constantly figuring out what the mental states of the other are.

#### A thick notion of perception

Interactionism embraces insights from enactivism, which is the view that cognition first and foremost consists of actively relating with the environment (see e.g., McGann and De Jaegher, 2009). From an interactionist point of view, social perception is closely related to dispositions for social interaction. This is in strong contrast to standard cognitivist approaches that tend to reduce human cognition to processes taking place inside the human brain, while the brain is in turn modeled as a “biological computer” manipulating with representations passively received from the “outside world.” Hence the concept of perception employed by interactionists is also different from the concept of perception that defenders of ToM use, and draws on e.g., the work of Merleau-Ponty (1945) and Gibson (1979).

While advocates of the ToM approach do not specify their concept of perception, they seem to have a rather narrow notion of it, given that one of their core assumptions is arguably that the minds of others are imperceptible. For interactionists, in contrast, perception is one of their core concepts, being rich both in content and function: perception does most of the work needed to understand and interact with others^8^.

#### Embodied minds and perceivable mental states

What about the interactionist understanding of mind and mental states? It is not easy to give interactionist account of them, as these notions do not seem to inherently belong to interactionist vocabulary. However, interactionists often use these concepts in order to enter into a dialog with the proponents of ToM, or, more broadly, with analytic philosophers of mind and cognitive psychologists. In contrast to ToM, interactionists claim that the mental states of others are not things which are completely hidden away or of a solely theoretical nature. The upshot is that in most everyday social encounters there is little need to figure out, either explicitly or implicitly, either via folk psychological theory or via simulation, what the hidden mental states of others behind their behavior are (Zahavi, 2007). We do not normally perceive others as mindless bodies moving around in physical space, but rather as persons, mind-body wholes, acting in concrete social environments (Zahavi, 2007). One prominent version of the interactionist thick notion of perception is the so-called direct perception thesis of social cognition (cf. Gallagher, 2008b), which states that we can directly perceive some of the mental states of others (such as emotions or intentions). Interactionists also criticize ToM for paying too little attention to context and to embodiment of social agents (Gallagher, 2001; Stawarska, 2006). For explaining social cognition, it is not enough to find out what is going on in the brains, because what is going on in the brains depends on what is going on in the bodies as well as in the physical and social environments where the bodies are situated^9^.

#### The affective dimension of social cognition

According to ToM (especially TT) our epistemic attitude toward others in its presumed third-person observational format is similar to the attitude of scientists toward their research objects. Interactionists, in contrast, emphasize the importance of affect and engagement in interpersonal interactions, which is assumed to give access for the participants to information that is otherwise much less obvious (Reddy and Morris, 2004, p. 657). Reddy and Morris even go so far as to state that “[e]ngagement creates the minds that are there to be known” (Reddy and Morris, 2004, p. 660), which is another way of pointing out that an emotionally rich relationship is qualitatively different and more fundamental to our everyday social life than a detached third-person ability to mindread. Although e.g. ST explains how we can automatically attribute emotions to other people, it fails to take into account the attributor's own emotional reactions as a source of information about the social situation. Our own affective responses often differ from the emotions that we recognize in others; for example, when I recognize anger in the other, I may become afraid. ToM can explain how I recognize anger in the other, but it does not account for how I become afraid as a result. According to interactionism, our affective responses play an important role for social interaction and they should be studied as a part of social cognition.

## ToM or interactionism? both!

### The blind side of radical interactionism

In general, interactionists have been rebelling against the monopoly of ToM over social cognition research. In doing so, some of them, that we refer to as *radical interactionists*, have insisted on a radical “interactive turn” in order to completely replace the framework of ToM with the new interactionist framework (see e.g., De Jaegher et al., 2010; De Jaegher, 2009a,b)^10^. It would thus appear that radical interactionists are falling into the same trap as ToM arguably faithfuls. They seemingly intend to monopolize social cognition research by suggesting to ignore the part of social cognition that is particularly human—the ability for abstract reasoning about other people's mental states.

At this point, it is crucial to distinguish between the different aims of social cognition research envisioned by interactionists and proponents of ToM. Asking the general question of how to explain human social cognition is different from the more specific issue of what individual cognitive (neural and psychological) mechanisms appertain to social cognition. According to interactionists, the advocates of ToM make the mistake of equating these two questions. At the same time, radical interactionists seem to make a similar mistake by claiming that explaining social cognition is a matter of detecting and explaining the perceptual and supra-individual processes that constitute real life social interactions.

Some of the radical interactionists are also *radical anti-individualists*, downplaying the importance of individual mechanisms in social cognition. For example, De Jaegher et al. (2010) argue that the interaction process itself can be seen as an enabling and constitutive factor for social cognition and should thereby be given an explanatory role in the theory. They go as far as to state that “[…] we can conceive of interaction dynamics as, in some cases, delivering the necessary cognitive performance. There is no need to duplicate their effects by an individual mechanism” (De Jaegher et al., 2010). It is not obvious what the statement amounts to, but we interpret it as a claim that individual explanations become superfluous once the interaction process is explained on the supra-individual level. We agree that social interaction is not reducible to the individual neural or cognitive processes; however, individual processes still remain a part of the whole story. We propose that a more promising approach would be a multi-layered account of a mechanistic nature (see Bechtel, 2008). It would enable us to give explanations on different levels (sub-personal, personal/individual, supra-individual)^11^ instead of arguing in favor of reductionism either of a sub-personal, individualist, or supra-individualist kind. It would also require the study of the links between the different levels of explanation.

Another vulnerable aspect of interactionism is that it tends to over-emphasize social interactions that are honest, smooth, and cooperative. It might well be the case that in situations where social interaction evolves smoothly in the direction that is agreeable for all participants, there is little need for the participants to bother with attributing mental states to others based on either simulation or inference. But it is questionable whether this is the case in situations of competition, disagreement, conflict, or obvious misunderstandings. It is surely possible that people sometimes observe other people from a purely third-person point of view and, moreover, attribute mental states to others to gain a better understanding of what they are up to.

Also, it is hardly possible to expand the characteristic of perceptibility to all mental states as it is obvious that some mental states (e.g., thoughts that are not expressed) are less perceivable than others (e.g., strong emotions), even by interactionist's standards. At times we do indeed find ourselves trying to figure out those more hidden aspects of others' mental lives. In addition, it is doubtful that interactionist approaches are able to account for many linguistic forms of interaction (e.g., those requiring Gricean assumptions about communicative intentions, see Grice, 1968, 1969), which is a reason to call for a ToM-like complement^12^.

To conclude the subject, a theory of social cognition should be able to account for both, the more direct forms of social interaction where no mindreading seems to be necessary, and the more detached and sophisticated forms of social understanding that require mindreading.

### Why is a complementary account needed?

Recently, a small number of authors have provided different arguments in favor of merging elements of ToM and interactionism instead of preferring one account over the other (Gangopadhyay and Schilbach, 2011; Michael, 2011). Our purpose is to expand upon this general idea in order to tease out new hypotheses for empirical research, drawing on dual process accounts of social cognition.

We have tried to demonstrate that while ToM is an individualist and a mentalizationist approach, interactionism is in many respects its antipode in preferring explanations in terms of embodiment, perception, and supra-individual processes. It seems, however, that ToM and interactionism may describe different aspects of social cognition. The important issue is therefore not asking whether ToM or interactionism is right, but rather asking what aspects of social cognition do these approaches capture and how are these different aspects related.

Interestingly, it seems that both ToM and interactionism aim to give alternative explanations to the same empirical findings (e.g., developing alternative accounts of the function of mirror neurons or interpreting developmental studies differently), but often they also refer to different empirical studies (cf. e.g., Goldman, 2006 and Reddy, 2008). This suggests that perhaps both approaches are right and wrong at the same time—right in some of what they state and wrong in some of what they deny. The important but difficult task is to figure out those elements from each approach that have got some aspect of social cognition right, and to see how they relate to elements that other approaches have captured. The way we see it, interactionism is looking at aspects of social cognition that seem to be more basic for social cognition, both phylogenetically and ontogenetically, and that we possibly share with other species. ToM concentrates on higher and specifically human traits of social cognition. It is as if these two sides focus on different layers of human social cognition. And indeed, there are several dual models of the human mind that have the potential to accommodate the insights of both, ToM and interactionism. We will turn to these in the next section.

## The dual models of cognition: type 1 and type 2 processes

### Dual process models in social psychology and in social cognitive neuroscience

Many everyday tasks in our lives require high speed and effectiveness of processing on the one hand, and great flexibility together with executive control on the other. Social interaction is no exception. Empirical evidence demonstrates that these characteristics do not fit well together, so how can coping in these tasks be explained? We favor a dual process approach to social cognition^13^.

Several recent cognitive models for social cognition in psychology and social cognitive neuroscience contrast two different processing types labeled e.g., lower level vs. higher level (Apperly, 2011, see also Apperly and Butterfill, 2009), automatic vs. controlled (Adolphs, 2009), implicit vs. explicit (Frith and Frith, 2008), pre-reflective vs. reflective (Keysers and Gazzola, 2007), low level vs. high level (Goldman, 2006) or reflexive vs. reflective (Lieberman et al., 2002; Lieberman, 2003).

The existence of numerous differently labeled but analogous dual process accounts is partly the result of the fact that many of them have been proposed in narrow fields of research independently of each other (e.g., in cognitive psychology and in social psychology, see Evans, 2010) and partly a result of every author's individualistic aim to offer an account that can be differentiated from other similar accounts. For the purposes of building a general integrative theoretical framework, we refrain from preferring one particular dual process theory over others and concentrate on the core of what these theories have in common. As Evans (2012) has pointed out: “there is [currently] both a broad consensus about the basic distinctions as well as lively debate about the specific nature of the two kinds of processing.” We use the terms “Type 1 processes” and “Type 2 processes” as general labels for the two types of sub-personal processes outlined by different dual process models. Other labels, such as “System 1/System 2” (Stanovich, 1999) or “intuitive mind/reflective mind” (Evans, 2010), have also been used for speaking about dual processes in general, but “Type 1/Type 2” seems to be the most neutral choice of terms and is therefore preferable, as Evans (2012) and Stanovich and Toplak (2012) have also pointed out. It is unlikely that the two types of processes map on only two cognitive systems (see Evans, 2008, and Keren and Schul, 2009), which is why we speak about two types of processes instead of two cognitive systems.

Type 1 processes are typically described as fast, efficient, stimulus-driven, and relatively inflexible. They place hard constraints on what information is considered or how it is processed to gain high efficiency and speed at the expense of low flexibility. These processes are thought to be closely linked with affect and their outcome is usually experienced as perceptions or feelings. Several authors have also pointed out that Type 1 processes might be evolutionarily older and shared with other species. Type 2 refers to a type of information processing that is relatively slow and cognitively laborious, making the opposite trade-off by being relatively flexible. The Type 2 processes typically involve some combination of effort, intention, and awareness. The processes tend to interfere with one another, and their outcome is sometimes experienced as self-generated thoughts. The Type 2 processes are thought to be responsible for explicit reasoning and to have no direct link with emotion. As such, they are considered to be evolutionarily more recent and uniquely developed in humans. (See e.g., Chaiken and Trope, 1999; Evans and Frankish, 2009; Evans, 2010; Apperly, 2011).

The typically correlated characteristics listed above should not, however, be considered as necessarily co-occurring features (Stanovich and Toplak, 2012). The dual process theoreticians have recognized the need to go beyond simply listing various dichotomous characteristics and emphasize the importance of finding operationalizable defining features for distinguishing between the two types of processing. There is more than one promising proposal on the matter. Evans (2008) has suggested that “Type 2 processes are those that require access to a single, capacity-limited central working memory resource, while Type 1 processes do not require such access.” Stanovich and Toplak (2012) have recently come up with a slightly different proposal, claiming that “autonomous processing is the defining feature of Type 1 processing” and “the key feature of Type 2 processing is the ability to sustain the decoupling of secondary representations.”

It is beyond the scope of this paper to give a fair review on the wealth of the dual process accounts and their critiques^14^, but several detailed overviews are available (e.g., Lieberman, 2007; Evans, 2010). In the following, we will focus on some of the most important features of the two types of processing that have been frequently emphasized in connection to social cognition.

### Mirrors and mentalizing

Dual process accounts of social cognition have emerged independently in the social neurosciences. Recent neuroscientific research suggests that the understanding of others' intentions is supported by two neural systems that perform complementary roles; *the mirror neuron system* (MNS) and *the mentalizing* or *theory of mind system* (ToMS). The MNS is thought to support the direct understanding of the intentions of the actions of others (Rizzolatti and Sinigaglia, 2010). In the human brain, the MNS is often associated with the intraparietal sulcus (IPS) and premotor areas in the inferior frontal gyrus (IFG). Based on the initial findings that the MNS is activated when performing actions and when observing same kind of actions performed by another individual, it was viewed as support for implicit forms of ST (Gallese and Goldman, 1998; Goldman, 2006). However, as discussed below (section “Interactionism vs. Implicit ToM), there are good reasons to question this interpretation. Alternatively, interactionism suggests that these processes are perceptual in nature. We will not try to resolve these issues in the present paper, but we do want to point out that regardless of whether these processes represent implicit simulation or perceptual processes, most will agree that the processes associated with the MNS are highly automatic, fast, and pre-conscious, thus fitting the broad description of Type 1 processes.

Although the MNS is thought to underlie the understanding of fairly complex motor intentions, it also has its limitations. For instance, Rizzolatti and Sinigaglia claim that “[u]nderstanding the reasons behind an agent's motor intention requires additional inferential processes” (2010, p. 271). Whilst we can directly grasp that someone intends to pick up a book from the table, the MNS is not thought to provide us with an understanding of the reasons that underlie that motor intention (for example, the wish to read the book, to put it back on the shelf or to bring it back to the library).

Generally, this distinction in action understanding corresponds to the difference between *proximal* and *distal* intentions, terms that are typically used in philosophy of action (Pacherie, 2008). It is generally believed that inferring distal intentions involves the activation of the ToMS, which is associated with a different set of cortical areas, minimally including the temporal parietal junction (TPJ) and the medial wall of the prefrontal cortex (mPFC) (Amodio and Frith, 2006; Frith and Frith, 2007). These areas have shown to be associated with several tasks that involve inferring intentions of others from a third person perspective (e.g., understanding cartoons or stories). Neuroimaging data does not provide further insight into what type of processing takes place in this network, but it can be hypothesized that this network subserves Type 2 mindreading processes. Within the social neurosciences, the ToM network is often associated with the explicit type of inferential processes, thereby fitting the broad description of Type 2 processes. In sum, these two systems fit the general division of Type 1 and Type 2 processes^15^.

Our aim is not to give an exhaustive overview of all processes or brain areas involved in social cognition (for extensive reviews see Lieberman, 2007; Rilling and Sanfey, 2011); however, we do want to point out two additional networks that have been consistently reported in social neuroscience research and are important for the current discussion. Firstly, the non-primary sensory areas in the posterior superior temporal sulcus (pSTS) and the fusiform gyrus (FFG). Human and animal studies have associated the pSTS with the initial sensory analysis of social signals, such as gaze direction (for review see Nummenmaa and Calder, 2009). The FFG, or specifically the fusiform face area (FFA), is thought to support the recognition of face identity. The output of these processes in these areas is thought to be input for both the MNS and the ToMS. Secondly, recent developments in social neuroscience have built upon the idea of *shared representations* in the MNS and extended this notion to mechanisms for understanding others' emotions (Decety, 2010). Indeed, numerous studies have shown that similar or overlapping brain areas, such as the insula, are activated when processing one's own and other people's emotions (Singer, 2006). Importantly, similarly to the models of motor resonance, theories of affective resonance claim that these processes are quick, automatic, and support a direct way of understanding other people's emotions (Decety, 2010).

## Joining theory of mind and interactionism to study dual processes

### The parallel between interactionism vs. ToM discussion and the type 1 vs. type 2 models of cognition

In this section we argue that there is a viable parallel to be drawn between the interactionism vs. ToM discussion and the Type 1 vs. Type 2 process models of cognition and that a promising way for enhancing research on social cognition is to consider interactionism as emphasizing those aspects of social cognition that pertain mostly to Type 1 processes, while considering ToM as emphasizing aspects that typically characterize Type 2 processes.

Interactionism stresses the perceptual and the affective dimension of social cognition. There is a clear parallel here to Type 1 processes. It is often stated in the dual process models that the outcome of Type 1 processes is usually experienced as perceptions, feelings, or emotions, whereas we have little conscious control over the processes that lead to these outcomes. The “intuitive mind” is considered to be shared across species and it is thought to be both phylogenetically and ontogenetically older and therefore more basic than the “reflective mind” (Evans, 2010). Interactionism resonates with these aspects by claiming to address those more primitive and presumably more fundamental aspects of social cognition that are largely ignored by the ToM approach.

On the other hand, standard accounts of ToM aim to find out what makes human social cognition distinctively human (see e.g., Saxe, 2006; Penn and Povinelli, 2007). This links directly to Type 2 processes as they are considered to be evolutionarily more recent and likely to be uniquely developed in humans (see e.g., Stanovich, 1999; Evans, 2010). Proponents of ToM concentrate on studying higher cognitive capacities such as meta-representation of other people's mental states (e.g., by standard false-belief tasks) of which non-human animals are not capable^16^. Unlike Type 1 processes, the outcome of Type 2 processes can sometimes be experienced as self-generated thoughts, whereas the phenomenology of explicit mindreading is much closer to reasoning than to perception. In sum, ToM, especially the explicit versions of it, points out traits of cognitive processing that are typically ascribed to Type 2 processes.

The connection between interactionism and the Type 1 processes may be more difficult to grasp. Earlier we criticized the tendency of the “radical interactionism” to prefer supra-individual descriptions over sub-personal explanations where actually both are needed. If interactionists lack sub-personal explanations for social cognition then the connection between Type 1 processes and interactionism is not obvious—after all, Type 1 processes are processes described on the sub-personal level. Most of the interactionists, however, do agree that a sub-personal story of the processes underlying social perception needs to be given as a part of social cognition theory. We argue that interactionism has potential to enrich our understanding of the Type 1 processes. Firstly, although the explanations given by ToM may seem plausible for explaining the functions of the Type 2 processes, the interactionist critique gives good reasons to doubt that the same explanations are suitable for the Type 1 processes. Secondly, interactionism provides detailed personal-level descriptions of social perception which can be used as proper scientific *explanandum* for the corresponding sub-personal explanations. Thirdly, interactionism describes supra-individual factors (environment, context, temporal dynamics of the interaction, embodiment, etc.) that is thought to facilitate social interaction. From these descriptions, it is possible to derive scientific variables that can be used in designing experiments for studying the effects of the “factors outside the skull” on real life social interactions. We hypothesize that many such variables have an effect particularly on the Type 1 processes, whereas Type 2 processes are less directly dependent on the current situation and the immediate surroundings.

### Interactionism vs. implicit ToM

In relation to the parallels outlined in the previous section, the question arises: what reasons are there to think that the Type 1 processes should be considered in terms of interactionism rather than in terms of implicit ToM? Implicit versions of ToM state that mindreading via theorizing or simulation takes place automatically and tacitly on the sub-personal level of processing. Some proponents of ToM have made a distinction between two kinds of mindreading which is similar to the differentiation between Type 1 and Type 2 processes. For example, Alvin Goldman (2006) distinguishes between high-level and low-level mindreading, both of which rely on some form of simulation of others' mental states (for critique of the distinction between two types of mindreading, see De Vignemont, 2009). This refers to the possibility that some types of mindreading might be implemented by Type 1 processes. Though our aim is not to settle this issue here, we would like to highlight some indicators against ToM accounts (ST in particular) of Type 1 processes.

Firstly, the concept of simulation involves some specific implications. For example, speaking of mirror neurons in terms of simulation implies that there is a primary and a secondary function for the mirror neurons. It is usually assumed that the primary function for mirror neurons is to contribute to the person's own actions and the secondary function is to simulate the actions of another “off-line”. However, it is possible that the activation of mirror neurons is not person-specific, so it has only one function; namely, to represent (a type of) an action as such. Information about who is the agent of the action may be “tagged” to this information in a later stage of processing. (see De Vignemont and Fourneret, 2004; Green, 2012).

Secondly, even if the use of the concept “simulation” were a good way for speaking about the mirror neurons, this does not automatically guarantee the success of any form of ST in a strict sense. ST traditionally implies much more than simply using the concept of simulation for some sub-personal processes that matter for social cognition. In principle, one can loosely use the concept of simulation for the mirroring processes without being a proponent of any form of ST in a strict sense^17^^,^^18^.

Finally, even if the function of mirror neurons were best described by ST, the study of the role of these neurons for social interaction still requires a wider interactionist methodology. In addition to the observational experiments that have been dominating the field within the ToM framework, mirror neurons and other cognitive and affective processes need to be studied in real life situations and in interactive contexts, where interactionists have argued that specific environmental and contextual factors may be important for social cognition.

Overall, we argue that it is highly unlikely that all aspects of social cognition can be explained in the framework of ToM, which focuses on the issue of how we attribute mental states to others. It is more parsimonious and evolutionarily more plausible to think that not all aspects of human social cognition rely on mental state ascriptions and to consider the capability for mental state ascriptions to have evolved from other, more primitive social abilities that we share with our evolutionary ancestors. ToM was initially proposed as an account for deliberate reasoning about other individual's mental states. As it became clear that people do not constantly reason about others' mental states, which made explicit versions of ToM unlikely to be correct as general theories of social cognition, most of the mindreading was hypothesized to happen implicitly. Although it is possible that over time mindreading becomes habitual and carries on automatically on an implicit level, making implicit mindreading responsible for all aspects of human social cognition is an evolutionary non-starter, as it leaves a huge gap between human and animal social cognition.

All these trains of thought suggest that one should be at least careful to associate mirror neurons with the ST exclusively and to consider ToM as an approach that is able to explain all aspects of human social cognition. Alternative approaches to Type 1 processes of social cognition must be considered, especially those that interactionism has a potential to offer.

To sum up, our basic claim is that there is a coarse-grained mapping between the Type 1 processes and interactionism on the one hand, and the Type 2 processes and ToM on the other. We do not expect this mapping to be clean and perfect—undoubtedly ToM in passing mentions aspects of social cognition that can be related to Type 1 processes and vice versa—but in general the tendencies are rather clear. On the example of how dual processes operate in a single brain and complement each other, one can think of ToM and interactionism as complementary theories of social cognition in general. The aim of proposing the new integrative framework is, however, not merely to dissolve the ToM vs. interactionism opposition, but to generate new research questions and hypotheses that would help to gain new knowledge on social cognition and especially on social interaction. In the following section, we outline a hypothesis on the relation of Type 1 and Type 2 processes.

## An integrative framework

Different dual process accounts for social understanding have been proposed by several authors over the past 10 years (e.g., Lieberman et al., 2002; Kilner et al., 2007; Keysers and Gazzola, 2007; De Lange et al., 2008). However, there is no consensus on *whether* and *how* these two types of processes cooperate and inform each other. Here we will review different positions commonly found in the literature, and argue that real life social interactions are rarely based on either Type 1 or Type 2 processes alone. On the contrary, we expect that in many cases of everyday social encounters both processes are simultaneously involved and that social behavior may be sustained by the interaction between these two types of processes.

### Independent networks

Some authors have suggested that the MNS and ToMS constitute independent networks and have complementary roles in social cognition (e.g., Saxe and Wexler, 2005; Jacob and Jeannerod, 2005; van Overwalle and Baetens, 2009). The general idea is that in most social situations we can rely on the set of low-level processes to support fluent and effortless social interactions. However, “[t]here seems […] to be a transition from the mirror to the mentalizing system […] when perceived body motions are contextually inconsistent, implausible, or pretended” (van Overwalle and Baetens, 2009). In line with interactionism, as presented by Shaun Gallagher (2008a), this means that the brain switches from MNS to ToMS when the situation appears puzzling.

Furthermore, van Overwalle and Baetens (2009) argue that current neuroscientific data supports the hypothesis that both systems are operating in isolation because most studies in their meta-analysis showed the involvement of only one of the two systems. Indeed, many studies have shown that in situations that call for an observational stance, for instance while reading a novel, we rely solely on the ToMS (Amodio and Frith, 2006). Similarly, there are many studies that have shown the sole involvement of the MNS in tasks involving imitating or passively watching simple movements. However, this only indicates that there are contexts in which these systems *are able to* operate in isolation. A possible explanation for the pattern of results reported in van Overwalle and Baetens' meta-analyses is that the experimental paradigms, and particularly the lack of real interactions, render MNS and ToMS to operate in isolation (see also Schilbach et al., forthcoming).

### Type 1 processes informing type 2 processes

A more common hypothesis in dual process models in general is that the Type 1 processes inform and support the Type 2 processes (e.g., Blakemore and Decety, 2001). This line of reasoning follows the logic of classic cognitivist models in which perceptual processes are temporally primary, and their outputs feed, uni-directionally, into cognitive processes. As such, the direct grasp of motor or proximal intentions (to switch a button) are used on a higher level to infer distal intentions (e.g., to turn on the light to read a book).

In line with this idea, some authors have suggested that the Type 1 processes associated with the MNS are a prerequisite for the ToM processes (e.g., Ohnishi et al., 2004). Given the neuroscientific evidence indicating that ToMS has often been found to operate in isolation from MNS, a strong version of this claim seems unlikely to be true. However, this does not refute weaker versions of this claim suggesting that Type 1 processes are an ontogenetic and/or phylogenetic prerequisite for Type 2 processes (Gopnik and Meltzoff, 1994; De Waal and Ferrari, 2010).

### Type 2 processes influencing type 1 processes

More recently, evidence has emerged in separate fields of research to support the idea that higher order (i.e., Type 2) representations regulate or bias perceptual (i.e., Type 1) processes. Several studies have already shown that higher order intentions can directly affect visual perception (Allport, 1987), for instance how we perceive tools is influenced by our intention to use them (Witt et al., 2005). More importantly, Teufel and colleagues (Teufel et al., 2009, 2010) have shown that mental state attribution (in this case the belief about whether the other person could see or not) also influences gaze-perception and automatic gaze-following. Based on these results, the authors hypothesize that perceptual modulation is the result of direct top-down modulation by the ToMS (Teufel et al., 2010). Additionally, others have suggested that the ToMS may also directly modulate the MNS (Liepelt and Brass, 2010; Ondobaka et al., 2012). For instance, Ondobaka et al. have shown that the motor congruence effect, or the fact that performing a movement is facilitated when another person is performing the same movement, is modulated by higher order mental state attributions. That is, they found that the movement-congruency effect was present only when participants acted with the same higher order intention as the co-actor. So in a similar fashion as our own proximal intentions (to pick up the cup) are often superseded and influenced by distal intentions (to get something to drink), our understanding of others also involves the top-down influence of higher order intentions. These findings are also in line with Jacob's alternative interpretation of the function of MNS which suggests that the MNS by itself is responsible not for the ascriptions of even the most simple intentions, but rather for computing “the motor commands from a representation of the agents prior intention” (Jacob, 2008); the intentions are thought to be computed by other means. On the other hand, Jacob also states that “the application of the concept of *grasping* triggered by the perception of an act of grasping would inferentially give rise to the related concept of, e.g., drinking” (Jacob, 2009), thus subscribing to the idea that the reciprocal interaction between these different systems may be essential in social understanding. In sum, these experiments also suggest that the engagement and top down influence of the putative ToMS is not only engaged when there is some incongruence in the social environment, but may also operate on whatever knowledge is available in order to make social interactions run more efficiently.

### Reciprocal influence

Taken together, the social neuroscience literature suggests that intentions are processed (and understood) at different levels of abstraction by different types of processes and that there are reciprocal interactions between these types of processing. Inspired by the predictive coding theory of the MNS (Kilner et al., 2007) we hypothesize that the MNS and the ToMS often operate in parallel; both function to enable predictions of behavior, which in turn facilitates social interactions. Furthermore, as is also suggested by dual process models, it is the interaction between different types of social cognitive processes that allows social behavior to be fast and effective, and at the same time flexible and context-dependent (Kilner et al., 2007; Evans, 2010).

Thus, although we fully agree that the “dynamic interaction between [distal] and [proximal] intentions modulates the processing of the observed actions of other people” (Ondobaka et al., 2012, p. 34), we also suggest that the processing of observing actions of others contributes to social interactions. Mindreading as implemented by the ToMs is therefore also in the service of interpersonal interaction (Jacob and Jeannerod, 2005). In this sense the view marries the ToM assumption that mindreading is important for social cognition with the interactionist assumption that social cognition is first of all social interaction.

**Figure 1:**
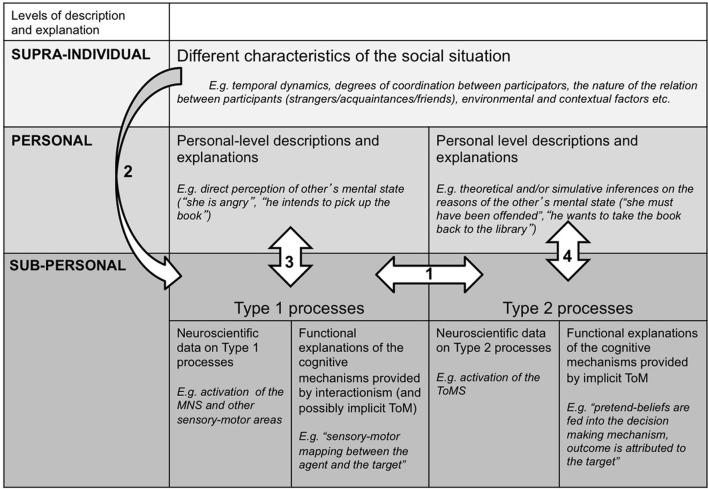
**An integrative account of social cognition**.

From this integrative account, two major conclusions for the interdisciplinary study of social cognition follow. Firstly, it follows that although it is possible to study processes emphasized by ToM and interactionism in isolation from each other, if we want to know how social cognition functions in real-life, we should rather study how these different processes influence one another. Secondly, in order to study the interaction between Type 2 (ToM) and Type 1 (interactionism) processes, we need to study actual social interactions and how these unfold over time. Figure 1 illustrates how ToM and interactionism can account for different aspects of social cognition on different levels of description and explanation. The arrows represent connections that we have outlined in the current paper: (1) the possible reciprocal influence between Type 1 and Type 2 processes on the sub-personal level; (2) the influence of supra-individual factors on Type 1 processes; (3) the correlations between Type 1 processes and personal level descriptions; (4) the correlations between Type 2 processes and personal level descriptions^19^. In the next section we consider several methodological implications for social cognitive neurosciences.

## Theoretical concerns become methodological concerns

Originally the field of social psychology was aimed at understanding how the behavior of people was influenced by the actual or imagined presence of others (Allport, 1985). The work focused mostly on individual behavior, but also developed paradigms to understand the relations between groups. Later, due to the cognitivist revolution, social cognitive processes also became a topic of study. However, the studies initially focused on how the individuals perceived others and rarely involved social interactions. In sum, actual social interactions and their specific dynamics were historically not a topic of study in the field of social psychology. Given this theoretical background, it is not surprising that the first social cognitive neuroscience studies did not involve studies on social interactions. As we have argued, there are several pressing theoretical arguments for studying social interactions. In this section we discuss some of the methodological issues that follow from the integrative framework.

### Social cognition in (inter)action

The interactionist critique addressing ToM is linked to a discussion over what empirical methods should be adopted for doing research on social cognition. Drawing on the discussions over the importance of “third-person” mindreading versus “second-person” engagement, interactionists insist that there is a need to overcome the “methodological solipsism” (Macmurray, 1991; Reddy and Morris, 2004) by complementing the purely observational methods with a “second-person” methodology (for a recent proposal on the matter, see Schilbach et al., forthcoming).

An interactive aspect of communication is in fact already inherent in standard “third-person” experiments. For example, before an experiment officially begins, the experimenter interacts with the test subject in order to explain what will happen and what is expected from the test subject. As Gallagher (2008a, p. 441) has pointed out, “even the youngest of the non-autistic children tested interact with the experimenter, and tend to understand what the experimenter wants them to do,” no matter whether they pass the false belief task or not. This seems to be a clear example of social understanding that goes without the full package of mindreading. Yet, for a long time, the presence of such social understanding remained largely unattended in social cognition research.

Such examples illustrate the interactionist claim that engaged social interactions are phenomenologically and cognitively different from passive observations of others. Although the suggestions for adopting a “second-person” methodology (experimenters interacting with test subjects) may seem too radical or not sufficiently rigorous to count as scientific, a less problematic way to improve the methodology is to find new ways for studying subjects while they are actively interacting during experiments rather than testing single persons who passively receive social stimuli. We hypothesize that studying social interactions in this manner will also allow us to learn more about the interaction between Type 1 and Type 2 processes. On many occasions, the division of labor between cognitive and social psychology has been drawn too sharply; both disciplines would gain from closer cooperation.

Within the neurosciences, social interactions have mostly been studied using game theoretical paradigms (for review see Rilling and Sanfey, 2011). Using these paradigms, neuroscientists have been able to gain a tremendous amount of knowledge on certain aspects of social interactions, such as the development of interpersonal trust (e.g., Delgado et al., 2005; King-Casas et al., 2005, 2008; van den Bos et al., 2009) and fairness-related behavior (e.g. Sanfey et al., 2003; Knoch et al., 2006; Guroglu et al., 2010). However, although economic games do capture some of the dynamics of social interactions, they arguably come as close to our everyday social interactions as correspondence chess—clearly lacking many essential features of everyday social interactions. Because of the rather detached stance and slow temporal dynamics of these interactions, it is not surprising that the neuroimaging data has revealed the consistent involvement of the mentalizing system in social interaction in economic games but, to our knowledge, never the involvement of the MNS.

More recently, several research groups have developed novel experimental paradigms that are specifically aimed at revealing the neural correlates of the processes underlying social interactions. One such study, designed by Schilbach and colleagues, involved an interactive gaze following paradigm (Schilbach et al., 2009). Interestingly, this study showed that, amongst other brain networks, the ToMS (MPFC) plays an important role in these real-time social interactions as well. Two other studies that involved real-time social interactions, one involving imitation (Guionnet et al., 2011) and the other the game of charades (Schippers et al., 2009), revealed the simultaneous involvement of the ToMS and MNS. As such, these results support the hypothesis that ToMS and the MNS are both involved in our everyday social interactions. However, these studies have not revealed anything about the possible interaction between the ToMS and MNS. In the next sections we will discuss several ways how we can build upon these initial findings of interactive paradigms.

### Interaction dynamics

The richness of social interactions that make controlled experiments so challenging also provides numerous new variables for experimental manipulation. Some examples include: the temporal dynamics of social interaction, degrees of coordination between participators, the nature, and history of the relation between participants (strangers/acquaintances/friends, etc.), the directness or mediation of contact, behavioral factors (body postures, gestures, eye-contact, etc.), experiential characteristics, or the mode of interaction (cooperative vs. competitive). For instance, cooperative vs. competitive social interactions are phenomenologically very different: cooperative interactions often feel effortless, whereas competition can be much more mentally taxing. To what extent do the relative contributions of Type 2 and Type 1 processes in cooperative vs. competitive contexts differ? What occurs when there is a shift from a cooperative to an antagonistic encounter? Or how might one's reputation (as a cooperator or non-cooperator) affect basic motor coordination processes? Although some of these questions can be answered using methods that have been traditionally used in the social cognitive neuroscience, the analyses of social interaction dynamics may require new methods, for example those used in dynamical systems analyses (De Jaegher, 2006, p. 87). For instance, simple oscillator models can be used to account for the potential coupling of physiological response patterns from two people during an interaction task (Helm et al., 2011). The couplings represent the degree of correspondence between the time series of both individuals, and this feature makes these models able to test hypotheses about physiological or behavioral interdependence within different types of relationships. As such, the novel variables generated by these models may reveal more about both social behavior and the underlying neural processes (For excellent examples of such novel interaction paradigms, see Auvray and Rhode, 2012; Lenay and Stewart, 2012, this issue).

### Connectivity

Another challenge is to understand the putative roles of the ToMS and MNS processes in social interactions, and importantly the interaction between these processes. As we have pointed out, previous imaging studies have revealed the involvement of both systems, but have provided little insight in their possible interaction. Here we think that connectivity analyses^20^ may reveal more about the interaction between different brain systems. Using different statistical methods, it is possible to discover whether there is stronger connectivity, or stronger correlation in patterns of activity, between different areas in the brain during different epochs of a social interaction. For instance, these methods might reveal that in the initial phase of a social interaction there is stronger coupling between the ToMS and MNS than in later phases of the interaction (or the other way around). However, given the correlational nature of these analyses, they are not able to inform us about the directionality of the supposed flow of information. Other, more sophisticated methods such as Dynamic Causal Modeling (DCM) or Granger Causality modeling (GC)^21^, allow for stronger inferences about the influence of one brain area on the activity of another (for overview of methods see Stephan and Roebroeck, 2012). Together these techniques may provide more insight in questions on the how and where of bottom-up biasing and top-down modulation of the MNS and ToMS, respectively. For instance, using these techniques we can figure out whether in some situations the ToMS directly modulates the pSTS, involved in processing biological motion (which then feeds into the MNS) or whether it directly modulates the MNS.

### Developmental perspective

Finally, developmental studies provide unique opportunities to see how the MNS and ToMS interact in ways that are not typical for adults when they are fully matured (De Haan and Gunnar, 2009; Decety, 2010). Recent research on the development of social cognition has shown that the components of the ToMS still show developmental changes until late adolescence (Blakemore, 2008; Burnett and Blakemore, 2009; Guroglu et al., 2011; van den Bos et al., 2011). Furthermore, one such study revealed that the connectivity within the ToMS also develops until late adolescence (Burnett and Blakemore, 2009). Taken together, this suggests that studies of the developmental changes in the interaction between the ToMS and the MNS may provide novel insights in the neurobiological mechanisms underlying social interactions. This will help us better understand the neural processes that underpin social interactions in adults and can also provide insight in the aetiology of developmental disorders such as autism.

## Summary

In this paper, we have characterized two rival approaches in the current social cognition research: the traditional *ToM* approach and the recently emerged, fast developing *interactionist* approach. Whereas ToM has been focusing on studying “third-person” mindreading (i.e., how people attribute mental states to others in order to explain and predict their behavior from a third-person point of view), interactionism insists on a radical turn toward focusing on “second-person” online interactions instead, claiming that the ToM accounts should be discarded in favor of more embodied and supra-individual explanations. We have argued that although it is common to view these two approaches as mutually exclusive, and indeed, they are to some extent based on contrasting philosophical assumptions, it is more fruitful to try to integrate them into a comprehensive theoretical framework instead. In order to develop new research questions and hypotheses from the integrative ToM-interactionist framework, we drew upon dual process theories of social cognition that contrast two different types of social cognitive processing. The first type (labeled *Type 1*) refers to processes that are fast, efficient, stimulus-driven, and relatively inflexible, and the second type (labeled *Type 2*) refers to processes that are relatively slow, cognitively laborious, flexible, and may involve conscious control. By comparing the ToM vs. interactionism debate with the dual process accounts, we proposed that interactionism captures types of social behavior based mostly on Type 1 processes, whereas ToM is more focused on those based on Type 2 processes. Furthermore, by suggesting that real life social interactions are rarely based on either Type 1 or Type 2 processes alone, we hypothesized that in most cases both processes are simultaneously involved and presumably also interact. Consequently, ToM and interactionism are relevant for studying different *albeit* related aspects of social interaction. Finally, we discussed some methodological implications derived from this new integrative framework, suggesting that studies in social cognitive neuroscience may benefit from investigating (1) the interaction of the Type 1 and Type 2 processes, and (2) developing experimental paradigms that are able to capture the dynamics of our everyday social interactions.

### Conflict of interest statement

The authors declare that the research was conducted in the absence of any commercial or financial relationships that could be construed as a potential conflict of interest.

## Footnotes

^1^We use the concepts *mindreading* and *mentalizing* interchangeably.

^2^The term *theory of mind* is used ambiguously in literature. In addition to the way we use it here, it is sometimes used to denote TT exclusively. In some contexts, the term *theory of mind* is used in a very broad sense to refer to an individual's ability to understand mental states, in others as well as in oneself.

^3^This implication does not hold for Gordon's simulation theory, however. See Gordon (1995, 2008).

^4^It does not mean that the only alternative to the unobservability thesis is behaviorism. Interactionists simply deny that there is a sharp boundary between observable behavior and mental states, which makes mental states completely “hidden” from perception. It is, however, debatable, whether giving up the unobservability thesis would change anything substantial for ToM, as the question of “what are the sub-personal mechanisms that enable us to perceive the mental states of others?” would still remain. Some proponents of ToM have even stated that their view is compatible with the idea of observability of mental states (e.g., Carruthers, 1996).

^5^Supra-individual factors are factors which either lie outside of an individual or are not reducible to processes inside of an individual, such as e.g., physical and social environment, context, temporal dynamics of the situation, etc. If pressed on this point, proponents of ToM would not deny that supra-individual factors play a role in social understanding, but according to interactionism, ToM has downplayed the importance of supra-individual factors.

^6^The methodological situation has started to change, presumably due to interactionist criticism. For instance, new “second-person” versions of the standard false-belief task have recently been developed where experimenters interact with test subjects in pragmatic contexts (see e.g., the study of Knudsen and Liszkowski, 2011).

^7^We distinguish between “radical” and “modest” anti-individualism. While “radical anti-individualism” is the view that once social cognition is explained on a supra-individual level, explanations on other levels become superfluous, “modest anti-individualism” insists that explanations on personal and sub-personal levels of explanation should be complemented by explanations on supra-individual level. On our account, all versions of interactionism are at least modestly anti-individualist, whereas some versions tend to be radical in their anti-individualism.

^8^See McGann and De Jaegher (2009) for an enactivist conception of social perception and Gallagher (2008b) for a direct perception thesis of social cognition.

^9^Some proponents of ToM, especially simulationists (e.g. Goldman, 2006), do consider the bodily aspects of others' behavior as important, but they view the observable bodily aspects of behavior as merely another type of input for mindreading. This approach is not satisfactory from the enactive and embodied point of view, which takes cognition to be much more deeply bodily structured. e.g., it is claimed that our sensory-motor system directly responds to others' behavior without any mediation via mentalizing.

^10^Less radical interactionists admit that in some cases (e.g., if the behavior of the other puzzles us) we may rely on simulation or theorizing, so they do not insist on ruling out the mindreading paradigm completely (see e.g., Gallagher, 2008b).

^11^In cognitive science it is a commonplace to distinguish between personal and sub-personal levels of explanation. This distinction goes back to Dennett (1969). The general idea is that the personal level deals with persons and their behavior and includes intentional vocabulary (e.g., “feels pain/intends to open the window/is angry” are personal level descriptions), whereas the sub-personal level deals with cognitive and neural mechanisms that serve different functions in a cognitive system and that can be described in a non-intentional vocabulary (e.g., “mirror neurons fire”). It makes sense to say e.g., “I feel angry” and “lateral orbitofrontal cortex is active” but it does not make sense to say “my brain feels angry” or “I activated my lateral orbitofrontal cortex.” Whereas explicit ToM operates on the personal level (it is the person who theorizes over or simulates the other person's behavior), implicit ToM serves as a middle ground between personal level and sub-personal neural level—it gives functional explanations on the sub-personal level in an intentional vocabulary (e.g., “mirror neurons are mechanisms for simulating the actions of another individual”). The exact relation between different levels is a much debated issue and it is not possible to give a detailed account on it here (but see e.g., Colombo, 2012 for a recent account on the matter). However, we assume that explanations on different levels should be considered as mutually informative. See also footnote 5.

^12^We are grateful to one of the reviewers for bringing this to our attention.

^13^Dual process accounts have been proposed by a great number of authors in a variety of cognitive domains, such as artificial intelligence, general reasoning, judgment, number cognition, memory, and social cognition. Here the discussion is restricted to dual process models of social cognition. In social psychology, the earliest models of dual processing date back to the 1980s; see e.g., Chaiken, 1980; Petty and Cacioppo, 1986; Chaiken and Trope, 1999.

^14^We want to highlight one issue that is often raised by critics of dual process models: the fact that when we learn certain skills (e.g., learn to drive), we seem to move from deliberative Type 2 processes toward more automatic Type 1 processes. This can be seen as a problem for dual process accounts, because it suggests either a gradient between Type 1 and Type 2 processes or a magical translation from Type 1 to Type 2 processes. The issue is beyond the scope of the current paper, but because of its importance, we address it briefly by drawing on the richer field of the neuroscience of learning. Numerous studies have shown that there are different learning mechanisms operating in parallel, and these mechanisms are associated with different brain systems (for an overview, see Balleine et al., 2008). Although these studies concern mainly very simple reward based learning paradigms, they might tell us something interesting about the learning of social skills. These studies make a distinction between goal oriented and habit learning systems. Whereas initially both systems are involved in behavior, after a certain amount of practice, the individual may rely on the habit system alone. Even though some skills can be learned by the habit system alone, other more complex behaviors need the initial presence of the goal-oriented system. Furthermore, relying on the habit system is possible only as long as the environment is very stable, e.g., if a person moves to another country, she might need to learn new social skills and inhibit the old ones. Although it may seem that during the learning process the behavior becomes gradually more automatic, this does not necessary imply that there is a gradient from Type 1 to Type 2 processes; it can just as well be the result of the relative contribution of Type1 and Type 2 processes. To what extent this line of reasoning applies to social skill learning remains an open question, which opens up some very interesting avenues for research in social neuroscience.

^15^Note that we use the processes associated with the MNS and ToMS as examples of Type 1 and Type 2 processes that are involved in social cognition, these are not meant to be considered exhaustive. For instance, other Type 1 non-MNS processes may exist, such as lower level perceptual processes.

^16^Chimpanzees and some other primates may be a possible exception. The issue of whether higher non-human animals are capable of some primitive form of mindreading has been a controversial matter since the end of 1970's and is far from being settled (cf. Premack and Woodruff, 1978; Call and Tomasello, 2008). However, even if e.g., chimpanzees can be considered to have some kind of ability for mindreading, it is certainly not as sophisticated as in humans.

^17^The same kind of argument can be used against TT. For example, claiming that social perception is inferential does not guarantee the success of TT.

^18^As one of our reviewers pointed out, one might argue that the two points about the concept of simulation could be met simply by adjusting one's definition of simulation. Our point is, however, that the interactionist critique of ST is not merely verbal—it is not reducible to the issue of whether one should use the term “simulation” or refrain from using it. Adjusting the definition of simulation to the extent that it would meet the above criticism would make the concept of simulation vacuous and would therefore rather weaken than strengthen the position of ST.

^19^The arrows on Figure 1 depict only those explanatory relations that are discussed in the current paper. The diagram allows to draw also other interesting connections (e.g., between supra-individual factors and Type 2 processes, or between supra-individual and individual level), but we do not discuss them here.

^20^Connectivity analyses are statistical methods that are aimed at understanding how the activity in one brain area (X) is related to another brain area (Y). One simple way of analyzing a possible relationship is to look at the correlation between time-series of areas X and Y. This is somewhat comparable of comparing how the index of different financial markets changes over time, and how the rises and falls of those indexes are correlated. In neuroimaging experiments, connectivity analyses are often aimed at testing whether the correlation between brain areas is affected by psychological states. Such analyses could for instance test whether there is a stronger connectivity between areas X and Y when someone observes a congruent or an incongruent action.

^21^DCM and GC are tools for testing different models of “causal” relationships between activities in multiple brain areas. As with other connectivity analyses, this involves the statistical analyses between time-series of different brain areas. The main idea is that a “causal” relation is assumed between X an Y when it is possible to predict changes in the time series of brain area Y based on what happens earlier in the time-series of X.
